# Injection time related to intraocular pressure using a CO_2_ driven preloaded injector: An experimental laboratory study

**DOI:** 10.1371/journal.pone.0254901

**Published:** 2021-07-19

**Authors:** Jan N. Weindler, Tadas Naujokaitis, Sonja K. Schickhardt, Ramin Khoramnia, Gerd U. Auffarth

**Affiliations:** Department of Ophthalmology, The David J. Apple International Laboratory for Ocular Pathology, University of Heidelberg, Heidelberg, Germany; Bascom Palmer Eye Institute, UNITED STATES

## Abstract

**Purpose:**

Experimental study to measure the intraocular lens (IOL) injection time and injection speed at different intraocular pressure (IOP) settings when using the AutonoMe® injector.

**Methods:**

In this experimental study, following phacoemulsification in porcine cadaver eyes, a trocar was inserted at pars plana with a connected infusion and IOPs of 20, 50 and 80 mmHg were generated by altering the infusion height. Twelve CO_2_ gas-driven injectors were used to implant an IOL via a corneal incision of 2.2 mm. For each IOP setting, the duration of the IOL injection and the injection speed was measured by analyzing a video recording of the procedure.

**Results:**

The mean ±SD injection time (seconds) was 4.47±0.50 at 20 mmHg, 4.98±0.55 at 50 mmHg and 5.47±0.20 at 80 mmHg. The mean ±SD injection speed (millimeters per seconds) was 1.36±0.15 at 20 mmHg, 1.22±0.14 at 50 mmHg and 1.10±0.04 at 80 mmHg.

There was a significant (p<0.05) difference between the 20 and 80 mmHg groups in mean injection duration and injection speed.

**Conclusion:**

The CO_2_ gas driven injector allows a safe IOL injection even at elevated IOP. Although the implantation time is slightly extended at higher IOPs, this does not seem to be clinically relevant. No IOL damage was observed at these pressure settings.

## Introduction

The development of injectors for lens implantation in cataract surgery has greatly contributed to reducing surgical trauma, by reducing the size of the corneal incision and shortening the surgical procedure time, while maintaining safety.

A recent trend is the introduction of preloaded injectors, simplifying lens handling, increasing safety and shortening the implantation time of the IOL [1,2]. In addition, the pre-loaded injector, lowers the risk of infection and toxic anterior segment syndrome by avoiding the use of reusable surgical instruments [3–5]. In the case of non-preloaded injectors, the IOL must first be inserted into the injector using forceps, a process prone to errors that can lead to damage of the lens or its incorrect insertion in the injector with consequent failure of implantation. Preloaded injectors omit this step and increase the safety of implantation.

Manufacturers try to improve the handling of the injectors further by simplifying their use. A screw propulsion mechanism allows a controlled and consistent implantation of the IOL, but both hands are required, one to hold the injector and the other to turn the screw. Injectors with a syringe-like mechanism require more surgical skill for implantation but can be operated with one hand. This is especially advantageous in difficult cases and/local anesthesia cases, where the second hand can then be used to stabilize the eye.

To minimize these risks, a new injector system for automatic injection of the IOL has been developed. The AutonoMe injector (Alcon Laboratories, Fort Worth, Texas, United States) uses CO_2_ gas pressure, operated by a speed control lever, to propel the lens out of the injector. Besides operating the lever, no additional force is required to inject the IOL by a one-handed, controlled implantation. In contrast to conventional injectors, it is not possible for the operator to use additional force to overcome resistance with a gas-driven injector. This is only ensured by pressing down the lever more strongly and thus using stronger gas pressure.

The IOP is normally between 9–21 mmHg [6,7]. During a routine cataract surgery, the IOP can fluctuate from 9 to over 400 mmHg [8–13]. Adding very small volumes into the eye greatly affects intraocular pressure [14]. Therefore, finding a reliable and consistent implantation of the IOL even with raised IOPs is necessary.

We investigated whether IOL injection is possible while using the CO_2_ gas-driven injector at three levels of IOP and assessed the influence of increased IOP on the new injector’s IOL injection time and injection speed.

## Materials and methods

### Experimental setup

In the present study, the use of animal organs complied with regional and national animal welfare laws. No testing was performed on living animals. No animals were sacrificed due to our tissue requirement. For this reason, in consultation with the responsible animal care commissioner of the University of Heidelberg, an ethics application was not necessary. The porcine eyeballs used in this study were obtained from white domestic pigs (Sus scrofa domesticus) immediately after slaughter from a local abattoir (Schradi Frischfleisch GmbH, Mannheim, Germany).

**Fig 1:**
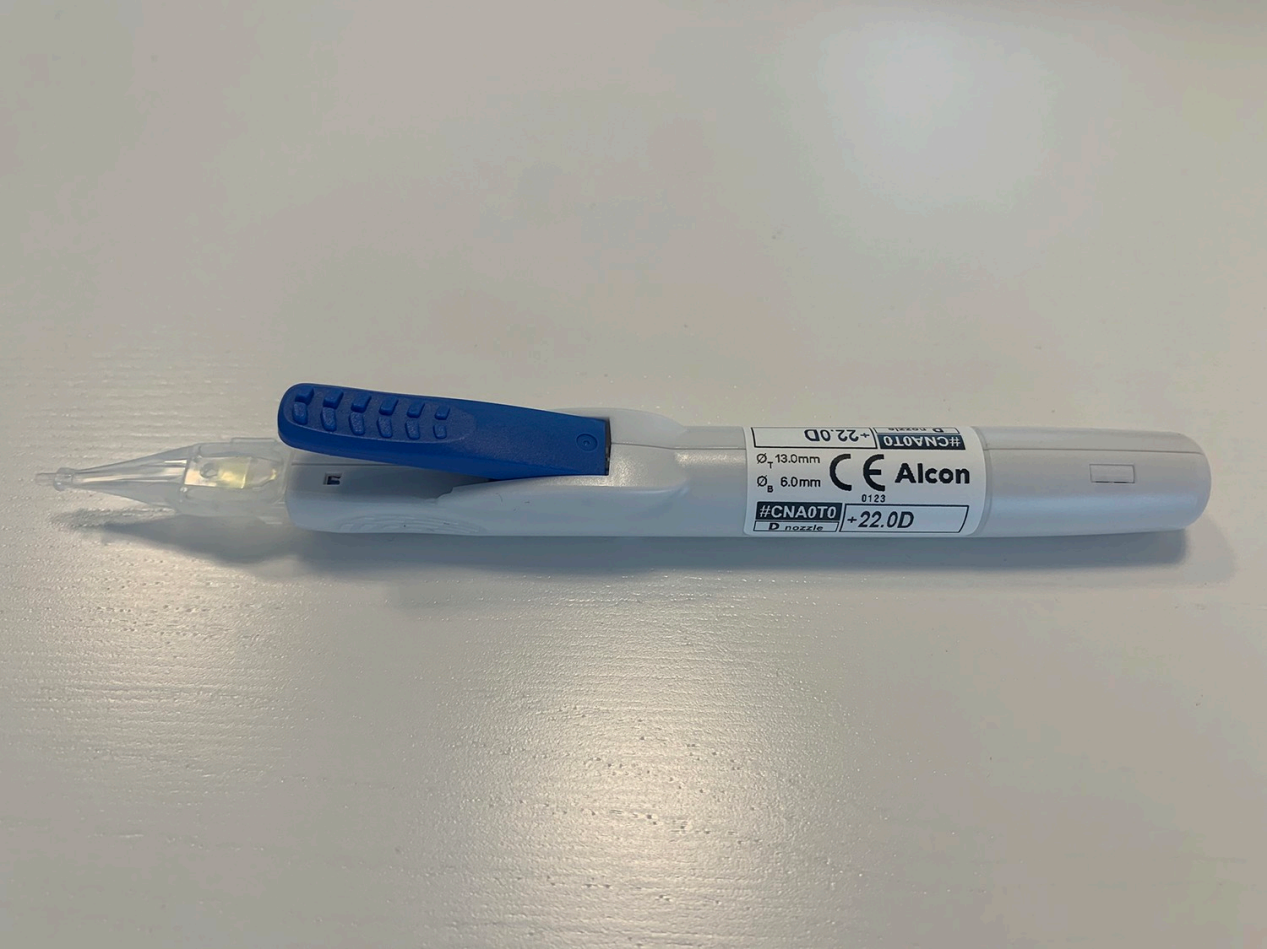
Preloaded AutonoMe® injector using compressed CO_2_ gas for propel the Clareon® IOL.

Twelve AutonoMe® injectors (Fig 1) and twelve porcine cadaver eyes were randomized in three groups, each group representing a target IOP value of 20, 50 or 80 mm Hg.

We used injectors already preloaded by the manufacturer with a +22 D Clareon® IOL (Alcon Laboratories, Fort Worth, Texas, United States) and the ophthalmic viscosurgical device (OVD) recommended by the manufacturer, ProVisc® (Alcon Laboratories, Fort Worth, Texas, United States).

**Fig 2:**
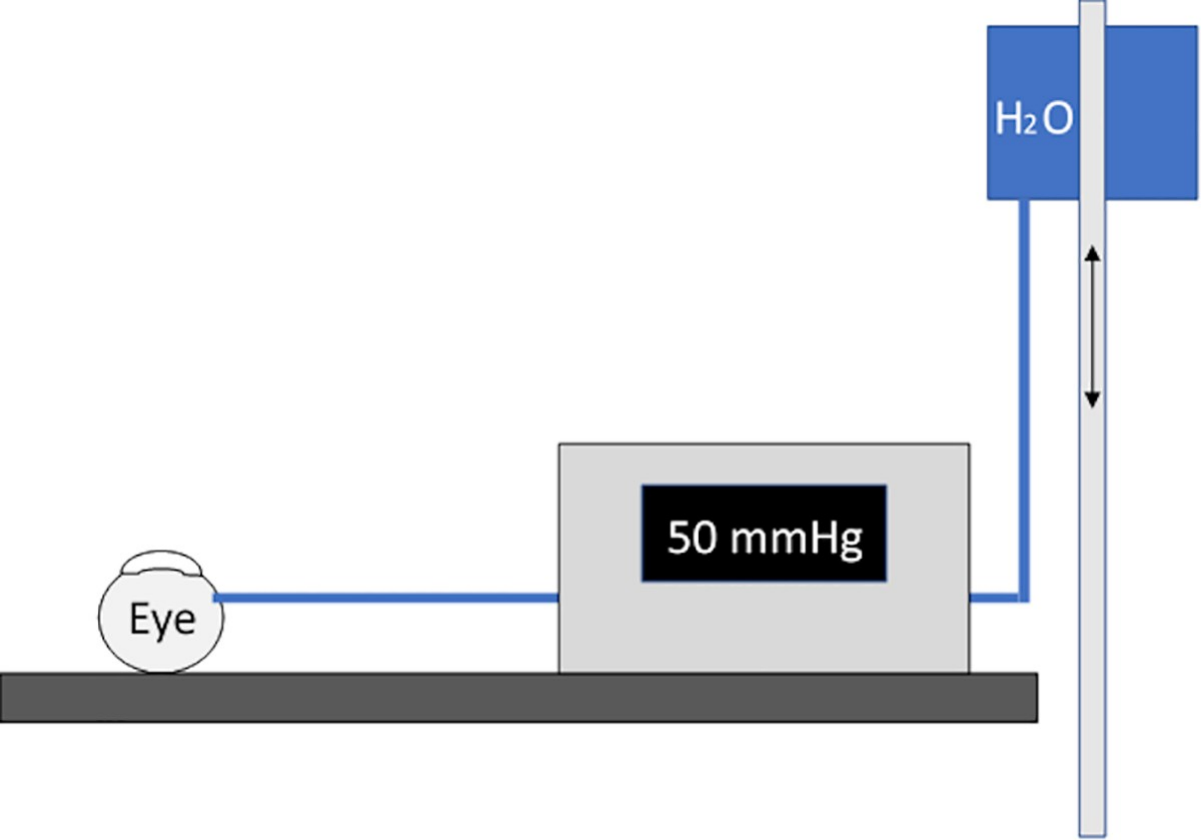
Setup: A ppv trocar was inserted pars plana into the vitreous cavity and connected to a height-adjustable infusion. By changing the height of infusion stand the intraocular pressure could be changed and checked with the vital data monitor.

A 2.2 mm clear-cornea incision and two paracentesis incisions were created, followed by the injection of OVD and formation of a capsulorhexis. Afterwards, hydrodissection, phacoemulsification and aspiration of cortex were performed using Megatron S3 (Geuder AG, Heidelberg, Germany). A pars plana vitrectomy (PPV) trocar was inserted into the vitreous cavity via the pars plana. A height-adjustable infusion was attached to the trocar and connected to the vital data monitor (IntelliVue MMS X2, Philips, Amsterdam, Netherlands) to measure the IOP (Fig 2). This approach, intravitreal measurement, was used to avoid the sensor having any influence on the lens implantation as it occurs in the anterior chamber. The IOL was advanced in the injector up to the mark, as shown in S1 Fig. and recommended by the manufacturer. The injector was then inserted into the anterior chamber through the 2.2 mm clear-cornea incision. By changing the height of the infusion stand, the desired pressure was generated and monitored using the data signs monitor. After the target pressure was reached, the infusion system was closed to prevent reflux of the infusion solution. Then the IOL was injected into the capsular bag by pressing the speed control lever down completely.

The IOL implantation was recorded with a video camera (Blackmagic Design Pocket Cinema Camera 4K, Blackmagic Design Pty Ltd., Port Melbourne, Australia). The implantation time and the injection speed were determined by video analysis. The IOL was first positioned in the injector tip. The starting point of the implantation was pressing the speed-control-lever and the end point was determined as the moment the trailing haptic left the injector tip completely. The injection of the IOL as well as the analysis of the videos was performed blinded, i.e. without the operator knowing the IOP. The examination and adjustment of the IOP was performed by an unblinded assistant. To determine the injection speed, the distance passed from the starting point at the injector tip to the opening of the injector was measured for 5 injectors. A distance of 6.0 mm was measured for all 5 injectors.

### Statistical analysis

The statistical analysis was performed using MedCalc software version 12.3.0.0 (MedCalc Software Ltd, Ostend, Belgium). The descriptive statistical analysis was performed for the injection time and the injection speed in the three IOP groups. Mean values, standard deviation (SD), median values and ranges were calculated. For the injection time, a test for normal distribution was performed for all 3 groups using the Shapiro-Wilk test and the visual inspection of Q-Q plots. Because the data were normally distributed, a parametric approach was chosen to assess the significance of differences between the three groups. A levene’s test was performed and equality of variances between the three groups was shown.

The ANOVA test was used to examine the mean values of all groups for statistical differences. A post-hoc analysis using Scheffé-test was then performed to compare the groups pairwise. The significance level was set at p<0.05.

## Results

**Fig 3:**
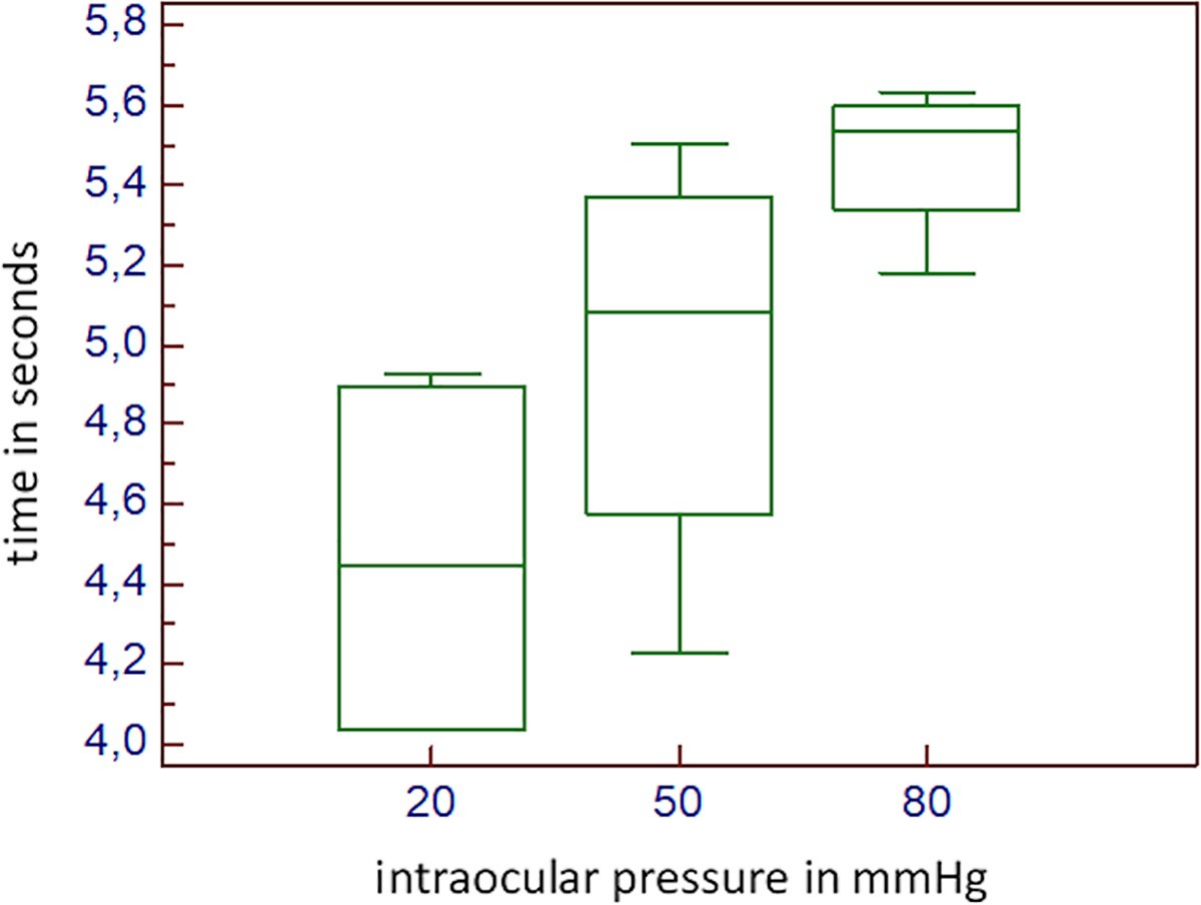
Boxplot of the injection time. The X-axis shows the three groups (20,50 and 80 mmHg) and the Y-axis shows the injection time in seconds [s]. Within the boxplot the median value is represented by the horizontal dividing line, and the top and bottom of the box represent the upper and lower quartile, with the whiskers indicating the maximum and minimum points.

Each IOL could be implanted without complications in all three IOP-groups. The mean (±SD) injection time was 4.47±0.50 s (seconds) at 20 mmHg, 4.98±0.55 s at 50 mmHg, and 5.47±0.20 s at 80 mmHg. The median values were 4.45 s (range 4.04–4.93 s) at 20 mm Hg, 5.08 s (range 4.23–5.50 s) at 50 mm Hg, and 5.54 s (range 5.18–5.63 s) at 80mm Hg (*Fig 3*).

The mean (±SD) injection speed calculated from the injection time was 1.36±0.15 mm/s at 20 mmHg, 1.22±0.14 mm/s at 50 mmHg and 1.10±0.04 mm/s at 80 mmHg. The median values were 1.36 mm/s (range 1.22–1.49 mm/s) at 20 mm Hg, 1.18 mm/s (range 1.09–1.42 mm/s) at 50 mm Hg, and 1.08 mm/s (range 1.07–1.16 mm/s) at 80mmHg (*Table 1*).

**Table 1:** Injection speed at 20, 50 and 80 mmHg.

IOP	20 mmHg	50 mmHg	80 mmHg
mm/s±(SD)	1,36±0,15	1,22±0,14	1,10±0,04

IOP = intraocular pressure, mmHg = millimeters of mercury, mm/s = millimeters per second.

An increasing IOP prolonged the IOL injection time and reduced the injection speed.

**Fig 4:**
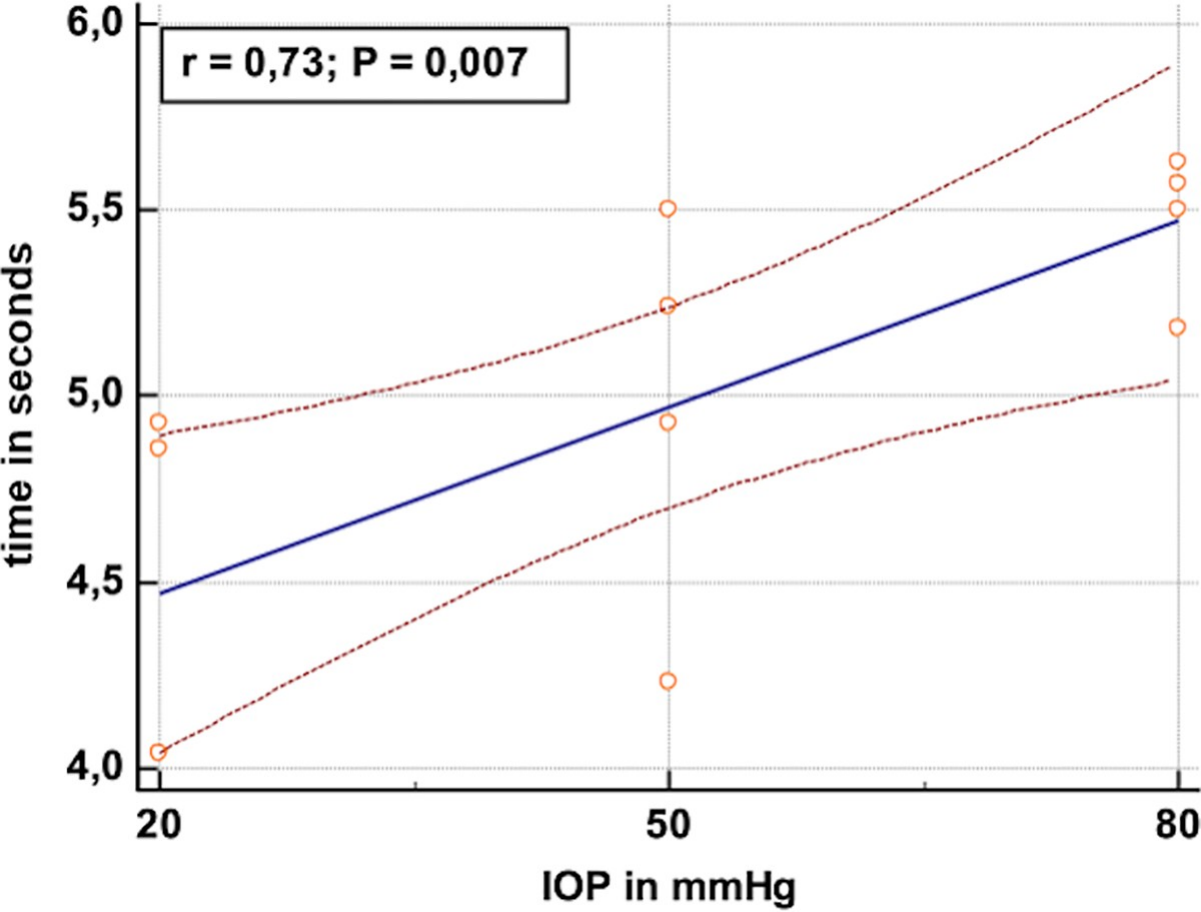
Scatter plot shows the injection time in seconds [s] (Y-axis) for each intraocular pressure [mmHg] (X-axis). Blue line: Regression line. Red lines: 95% confidence interval.

A statistically significant difference in injection time of approximately 1 second was observed between the 20 mm Hg and 80 mm Hg groups (p = 0.032). There was no statistically significant difference for the injection time between the 20 mm Hg and 50 mm Hg groups, as well as between the 50 mm Hg and 80 mm Hg groups (p>0.05). The relationship between IOP and injection time correlated significantly (p<0.007) with R = 0.73 (Fig 4). The relationship is approximately linear for the studied measurement points.

## Discussion

The AutonoMe injector utilizes a novel mechanism for advancing the IOL, by using compressed CO_2_ gas. Since fluctuations of the IOP occur even during routine cataract surgery, it is important to ensure that the injector allows a reliable IOL implantation also at high IOP.

In a recent study by Usui and Tanaka [15] different preloaded and non-preloaded injectors of the syringe type were compared in terms of the resistance force which has to be passed during implantation of the IOL. The study was able to show that there are big differences in the resistance force between the injector systems. Some injectors only had to pass a resistance force of 2.5 Newton and others up to 20 Newton during IOL injection. If a high level of resistance force has to be passed, then this increases the risk of injuring the IOL or intraocular structures during injection.

In contrast to other currently available IOL injectors, the injector assessed in this study uses CO_2_ gas to propel the IOL. In common with the syringe-action injectors, it can be operated with one hand, allowing the surgeon to better concentrate on controlling the IOL implantation.

It is important to ensure that this novel mechanism for advancing the IOL can successfully overcome resistance of high IOP, which is not uncommon during cataract surgery. Large pressure fluctuations between 9–400 mmHg occur during cataract surgery [8–13]. Short-term pressure peaks of up to 400 mmHg have been observed during hydrodissection. However, high IOPs were also measured during phacoemulsification and the IOL injection. Zhao et al. [8] used an intracameral pressure sensor in 25 patients during cataract surgery and found the IOP between 13 and 96 mm Hg. In another in vivo study, which included 9 patients, the IOP of 10–50 mm Hg was measured with the electronic applanation tonometer [10]. Khng et al. [9] conducted an experimental study in 3 cadaver eyes using a sensor in the vitreous cavity and found that the IOP was above 60 mm Hg 48–85% of the time during cataract surgery.

The IOP at the start of lens injection is between 18–55 mmHg depending on the study [9,10].

In this study we have chosen the three groups to test the injector for reliability at different IOPs. The IOP in group 1 and 2 with 20 and 50 mmHg is chosen as it occurs before implantation of the IOL [9,10] during cataract surgery. For group 3 a pressure of 80 mmHg was chosen to test the reliability of the injector at high intraocular pressure.

During the injection the IOP continues to increase and reaches values of up to 122 mmHg [9]. In the Khng group’s study [9] the IOP at the beginning of the injection was 55 mmHg. Such sudden changes in pressure can increase the risk of optic nerve disc cupping and can lead to an acute reversible impairment of the visual field, especially in glaucoma patients [16,17]. Therefore, a reliably uniform implantation is required.

Gotoh et al [18] examined experimentally the pressure changes of different injectors during injection of the IOL. They could show that the IOP increases up to 100 mmHg at a base pressure of 15 mmHg during implantation of the IOL. This strong pressure increase could be demonstrated for syringe-type injectors as well as for injectors with a rotating mechanism. The pressure curve for injectors with a rotating mechanism showed a rapid pressure increase and then a gradual pressure decrease. In the case of syringe-type injectors, a gradual increase in pressure was followed by a sudden drop in pressure.

In a recently published clinical study [19], the injection time of preloaded injectors was investigated. The AutonoMe injector showed an injection time of 8.6±1.9 seconds. In this clinical study the speed of the IOL injection was determined by the surgeon. This, in addition to the acquisition of other start and end points of the injection, explains the prolonged injection time compared with this experimental study, in which the IOL was injected at maximum speed. Light and electron microscopic examinations show that a CO_2_ gas-driven injector has little to no damage after implantation [19].

According to the manufacturer [20], the AutonoMe Injector should achieve a delivery speed of up to 3 mm/s. We recorded maximum injection speeds between 1.07 to 1.49 mm/s.

High initial pressure of 80 mmHg influenced the injection of the IOL and prolonged the implantation time. The range for all injections was 4.04–5.63 seconds. There was no prolonged injection time of clinical importance. All IOLs could be implanted without complications. Our results show that the AutonoMe injector ensures a reliable and undamaged implantation of the IOL even in conditions of high IOP.

## Supporting information

S1 Fig Preparation of the injector. The intraocular lens was pushed forward until the anterior haptic was at the mark.
(TIF)

S1 Table Results of all injections. (XLSX)

## References

[1] Chung B, Lee H, Choi M, Seo KY, Kim EK, Kim T-I. Preloaded and non-preloaded intraocular lens delivery system and characteristics: human and porcine eyes trial. International journal of ophthalmology. 2018;11(1):6–11. doi: 10.18240/ijo.2018.01.02 29375983 PMC5767650

[2] Jones JJ, Chu J, Graham J, Zaluski S, Rocha G. The impact of a preloaded intraocular lens delivery system on operating room efficiency in routine cataract surgery. Clin Ophthalmol. 2016;10:1123–9. doi: 10.2147/OPTH.S107726 27382245 PMC4918740

[3] Mayer E, Cadman D, Ewings P, Twomey JM, Gray RH, Claridge KG, et al. A 10 year retrospective survey of cataract surgery and endophthalmitis in a single eye unit: injectable lenses lower the incidence of endophthalmitis. The British Journal of Ophthalmology. 2003;87(7):867–9. doi: 10.1136/bjo.87.7.867 12812888 PMC1771777

[4] Mathys KC, Cohen KL, Bagnell CR. Identification of unknown intraocular material after cataract surgery: evaluation of a potential cause of toxic anterior segment syndrome. J Cataract Refract Surg. 2008;34(3):465–9. doi: 10.1016/j.jcrs.2007.10.047 18299073

[5] Toxic anterior segment syndrome after cataract surgery—Maine, 2006. MMWR Morb Mortal Wkly Rep. 2007;56(25):629–30. 17597694

[6] Leydhecker W, Akiyama K, Neumann HG. [Intraocular pressure in normal human eyes]. Klin Monbl Augenheilkd Augenarztl Fortbild. 1958;133(5):662–70. 13621563

[7] Wang YX, Xu L, Wei WB, Jonas JB. Intraocular pressure and its normal range adjusted for ocular and systemic parameters. The Beijing Eye Study 2011. PLoS One. 2018;13(5):e0196926–e. doi: 10.1371/journal.pone.0196926 29771944 PMC5957383

[8] Zhao Y, Li X, Tao A, Wang J, Lu F. Intraocular Pressure and Calculated Diastolic Ocular Perfusion Pressure during Three Simulated Steps of Phacoemulsification In Vivo. Investigative Ophthalmology & Visual Science. 2009;50(6):2927–31. doi: 10.1167/iovs.08-2996 19168897

[9] Khng C, Packer M, Fine IH, Hoffman RS, Moreira FB. Intraocular pressure during phacoemulsification. J Cataract Refract Surg. 2006;32(2):301–8. doi: 10.1016/j.jcrs.2005.08.062 16565009

[10] Hejsek L, Kadlecova J, Sin M, Velika V, Jiraskova N. Intraoperative intraocular pressure fluctuation during standard phacoemulsification in real human patients. Biomed Pap Med Fac Univ Palacky Olomouc Czech Repub. 2019;163(1):75–9. doi: 10.5507/bp.2018.065 30398219

[11] Grinbaum A, Blumenthal M, Assia E. Comparison of intraocular pressure profiles during cataract surgery by phacoemulsification and extracapsular cataract extraction. Ophthalmic Surg Lasers Imaging. 2003;34(3):182–6. 12757089

[12] Vasavada V, Raj SM, Praveen MR, Vasavada AR, Henderson BA, Asnani PK. Real-time dynamic intraocular pressure fluctuations during microcoaxial phacoemulsification using different aspiration flow rates and their impact on early postoperative outcomes: a randomized clinical trial. J Refract Surg. 2014;30(8):534–40. doi: 10.3928/1081597X-20140711-06 25325894

[13] Kamae KK, Werner L, Chang W, Johnson JT, Mamalis N. Intraocular pressure changes during injection of microincision and conventional intraocular lenses through incisions smaller than 3.0 mm. J Cataract Refract Surg. 2009;35(8):1430–6. doi: 10.1016/j.jcrs.2009.03.038 19631132

[14] Silver DM, Geyer O. Pressure-volume relation for the living human eye. Curr Eye Res. 2000;20(2):115–20. 10617912

[15] Usui M, Tanaka T. Resistance force for intraocular lens insertion through lens cartridges and syringe-type injectors. J Cataract Refract Surg. 2015;41(8):1745–51. doi: 10.1016/j.jcrs.2015.03.018 26432134

[16] Azuara-Blanco A, Harris A, Cantor LB, Abreu MM, Weinland M. Effects of short term increase of intraocular pressure on optic disc cupping. Br J Ophthalmol. 1998;82(8):880–3. doi: 10.1136/bjo.82.8.880 9828770 PMC1722706

[17] Trible JR, Anderson DR. Factors associated with intraocular pressure-induced acute visual field depression. Arch Ophthalmol. 1997;115(12):1523–7. doi: 10.1001/archopht.1997.01100160693005 9400785

[18] Norihito G, Matsushima H, Tadashi S. Changes in Anterior Chamber Pressure During Injection of Different IOL Types Through Microincision. ASCRS 2012 Poster on Demand; USA,Chicago 2012.

[19] Khoramnia R, Yildirim TM, Weindler J, Naujokaitis T, Dzhambazova M, Auffarth GU. Preloaded injectors used in a clinical study: videographic assessment and laboratory analysis of injector-nozzle damage. Journal of Cataract & Refractive Surgery.Publish Ahead of Print. 10.1097/j.jcrs.0000000000000587 34469394

[20] Nuijts R, Werner L, Auffarth G, Lane S. Clareon ®IOL: A New Monofocal Platform. ESCRS Portugal, Lisbon 2017.

