# Supplementary figures and images for: Injection time related to intraocular pressure using a CO2 driven preloaded injector: An experimental laboratory study
Source: PLoS One. 2021 Jul 19;16(7):e0254901. doi: 10.1371/journal.pone.0254901 (PMC8289067; doi:10.1371/journal.pone.0254901)

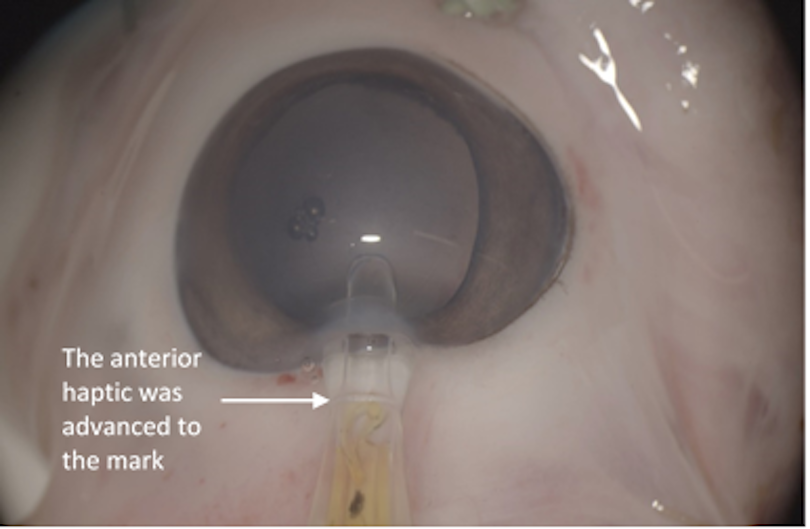

Supplement: S1 Fig — The intraocular lens was pushed forward until the anterior haptic was at the mark. (TIF) [file pone.0254901.s001.tif]
